# A novel signature derived from metabolism-related genes GPT and SMS to predict prognosis of laryngeal squamous cell carcinoma

**DOI:** 10.1186/s12935-022-02647-2

**Published:** 2022-07-08

**Authors:** Yujie Shen, Qiang Huang, Yifan Zhang, Chi-Yao Hsueh, Liang Zhou

**Affiliations:** Department of Otorhinolaryngology, Eye & ENT Hospital, Fudan University, Shanghai, 200031 China

**Keywords:** Metabolism, Prognosis, Tissue microarray, Laryngeal squamous cell carcinoma, Nomogram

## Abstract

**Background:**

A growing body of evidence has suggested the involvement of metabolism in the occurrence and development of tumors. But the link between metabolism and laryngeal squamous cell carcinoma (LSCC) has rarely been reported. This study seeks to understand and explain the role of metabolic biomarkers in predicting the prognosis of LSCC.

**Methods:**

We identified the differentially expressed metabolism-related genes (MRGs) through RNA-seq data of The Cancer Genome Atlas (TCGA) and Gene set enrichment analysis (GSEA). After the screening of protein–protein interaction (PPI), hub MRGs were analyzed by least absolute shrinkage and selection operator (LASSO) and Cox regression analyses to construct a prognostic signature. Kaplan–Meier survival analysis and the receiver operating characteristic (ROC) was applied to verify the effectiveness of the prognostic signature in four cohorts (TCGA cohort, GSE27020 cohort, TCGA-sub1 cohort and TCGA-sub2 cohort). The expressions of the hub MRGs in LSCC cell lines and clinical samples were verified by quantitative reverse transcriptase PCR (qRT-PCR). The immunofluorescence staining of the tissue microarray (TMA) was carried out to further verify the reliability and validity of the prognostic signature. Cox regression analysis was then used to screen for independent prognostic factors of LSCC and a nomogram was constructed based on the results.

**Results:**

Among the 180 differentially expressed MRGs, 14 prognostic MRGs were identified. A prognostic signature based on two MRGs (GPT and SMS) was then constructed and verified via internal and external validation cohorts. Compared to the adjacent normal tissues, SMS expression was higher while GPT expression was lower in LSCC tissues, indicating poorer outcomes. The prognostic signature was proven as an independent risk factor for LSCC in both internal and external validation cohorts. A nomogram based on these results was developed for clinical application.

**Conclusions:**

Differentially expressed MRGs were found and proven to be related to the prognosis of LSCC. We constructed a novel prognostic signature based on MRGs in LSCC for the first time and verified it via different cohorts from both databases and clinical samples. A nomogram based on this prognostic signature was developed.

**Supplementary Information:**

The online version contains supplementary material available at 10.1186/s12935-022-02647-2.

## Background

Laryngeal squamous cell carcinoma (LSCC) is a most common cancer of the upper respiratory tract. Smoking and drinking are well-recognized risk factors for LSCC [1]. The Global Cancer Observatory reported more than 170,000 new cases of laryngeal cancer and more than 94,000 deaths due to laryngeal cancer in 2018 [2]. Despite the continuous improvement in the treatment and management, the prognosis for LSCC patients remains unsatisfactory and the mortality rate is still high [3]. Most LSCC cases are in the locoregionally advanced stage at the time of diagnosis [4]. The recurrence rate is high and the patients often develop resistance to chemotherapy or radiotherapy. The clinical outcome for patients with advanced laryngeal cancer remains poor [5]. Hence, identifying a clinically tractable biomarker that can effectively predict the prognosis of LSCC would greatly benefit both the clinicians and the patients.

Metabolism, a series of reactions within cells of living organisms to sustain life, involves many interconnected cellular pathways to ultimately provide energy for various cell functions [6]. The current research on metabolism aims to solve biological problems from the level of molecular characters and results [6]. Recent studies have shown that metabolic reprogramming regulates oncogenesis and tumor development. Gong et al. have found that lipid metabolism reprogramming in cancer-associated fibroblasts potentiates migration of colorectal cancer cells [7]. In breast tumors, ADHFE1 and MYC signaling contributes to the accumulation of D-2HG, an oncogenic metabolite and potential driver of disease progression [8]. Tobacco smoking is shown to induce metabolic reprogramming in renal cell carcinoma [9]. These reprogramming activities can help to meet the bioenergy, biosynthesis, and redox needs of cancer cells, and thus are now considered as markers of cancer [10]. The expression of glutamic pyruvic transaminase (GPT, also known as alanine aminotransferase) can reflect the change of energy metabolism in the tissues of skeletal muscle, kidney, and liver [11]. In addition, GPT plays a critical role in the intermediary metabolism of glucose and amino acids, catalyzing the reversible transamination between alanine and 2-oxoglutarate to generate pyruvate and glutamate [12]. And its role in several cancers has also been proved [13–18]. There are two GPT subtypes in mammals, GPT1 and GPT2 [19]. GPT2 has been widely suggested as a critical factor for the tumorigenesis of various cancers [14, 18]. However, there are few reports about GPT1 in cancer. Whether GPT1 can regulate energy metabolism and tumor cell proliferation, remains to unclear. Spermine synthase (SMS) catalyzes the production of spermine from spermidine. The missense mutation of SMS may cause Snyder-Robinson Syndrome (SRS) [20, 21]. The overexpression of SMS can facilitate colorectal cancer cell growth [22], while the inhibition of SMS expression may be associated with TGF-β-induced growth inhibition in hepatoma cells [23]. Although metabolism in cancer has attracted more and more attention, little research has been done regarding the relationship between LSCC and metabolic markers.

In this study, we analyzed metabolomic profiling of LSCC patients and classified them into different groups. Through the mutual verification of different databases and tissue microarray (TMA), we developed and verified a novel prognostic signature based on metabolism-related genes (MRGs), which might eventually provide better LSCC patient classification and guide treatment from the perspective of metabolism.

## Methods

### Data acquisition

We collected RNA-seq data and microarray data to prove the effectiveness of this study. During the process of integrating The Cancer Genome Atlas (TCGA) database, we logged into the public database website (https://portal.gdc.cancer.gov/) [24] and set the following criteria: 1 “Repository”; 2 “Cases”; 3 “Primary Site: larynx”; 4 “Program: TCGA”; 5 “Files”; 6 “Data Category: transcriptome profiling”; 7 “Data Type: Gene Expression Quantification”. Eventually, RNA-Seq data originated from 123 LSCC samples, including 111 LSCC tumor samples and 12 matched normal samples, were downloaded and used for this study. The clinical information of 111 LSCC patients from TCGA is demonstrated in Additional file 7: Table S1. Also, we filtered the microarray data and downloaded GSE27020 dataset including 75 non-recurrent and 34 recurrent LSCC patients from Gene Expression Omnibus (GEO, https://www.ncbi.nlm.nih.gov/geo/) [25] for the subsequent verification of the prognostic signature [26].

### Identification of differentially expressed MRGs

We collected all the metabolic-related pathways and corresponding genes contained in the Gene set enrichment analysis (GSEA) database (Additional file 8: Table S2) [27]. The expression of metabolic-related genes was extracted from TCGA and differential expression analysis was conducted by “limma” [28] package in R software (version: × 64 3.6.1). We set the fold change (FC) equal to the expression of genes in LSCC tissues divided by the expression in normal tissues. The screening conditions were |log (FC)|≥ 1 and an adjusted *p*-value < 0.01.

### Pathway enrichment analysis and protein–protein interaction (PPI) analysis

In order to explore the biological functions of differentially expressed MRGs, we performed Gene Ontology (GO) [29] and KEGG (Kyoto Encyclopedia of Genes and Genomes) analysis [30]. Then, these genes were introduced into String database (https://string-db.org/) [31] to eliminate the genes with poor connectivity and the results were visualized through Cytoscape software (Version 3.7.1) [32].

### Construction and validation of a prognosis signature based on MRGs

We collected and downloaded RNA-seq data of 111 LSCC patients and the corresponding clinical information from TCGA database. After integrating the clinical information, we deleted 15 patients with unknown “stage” status and 6 patients with unknown “N” status. Finally, 90 LSCC patients with complete clinical information were enrolled. Univariate Cox regression analysis was performed to screen out prognostic MRGs. Least absolute shrinkage and selection operator (LASSO) analysis and multivariate Cox regression analysis were applied to construct a prognostic signature. The risk score formula of the prognostic signature was as follows: Risk score = coef * Exp (geneA) + coef * Exp (geneB) + coefi * Expi (genei) [33, 34]. The 90 patients were divided into high- and low-risk groups according to the median risk score (1.244). Kaplan–Meier survival analysis and receiver operating characteristic (ROC) curve were used to measure the reliability and validity of the signature as internal validation. The same analysis was applied in GSE27020 cohort as external validation to verify the effectiveness of the prognostic signature developed based on MRGs in predicting disease-free survival (DFS) of LSCC. Moreover, in order to further verify the universality of the prognostic signature, 90 patients in TCGA cohort were randomly divided into TCGA-sub1 and TCGA-sub2 cohorts in a 1:1 ratio, and the same analysis method was applied.

### Ethics statement and tissue specimens

After obtaining the approval from the Ethical Committees of Eye and ENT Hospital, Fudan University, a total of 62 paired samples from LSCC patients were collected between September 2020 and July 2021. LSCC and adjacent normal tissues, which were ≥ 3 cm distal to the incisal edge, were stored at − 80 °C and used for quantitative reverse transcriptase PCR (qRT-PCR). The informed consents were released by the LSCC patients with their agreement.

### Cell culture

The LSCC cell lines LSCC-31, FD-LSC-1, AMC-HN8 and Tu686 from LSCC patients were prepared and tested. LSCC-31 and FD-LSC-1 were cultured in BEGM (CC-3170 Lonza), Tu686 in DMEM (Gibco), and AMC-HN8 in RPMI-1640 (Gibco), with 10% fetal bovine serum (Gibco) at 37 ℃ in the presence of 5% CO_2_. LSCC-31 and FD-LSC-1 cell lines were obtained from our lab [35], while Tu686 cell lines was obtained from Cell Bank of the Shanghai Institute of Cells, Chinese Academy of Science (Shanghai, China). AMC-HN8 cell lines was a kind gift from Professor Kim SY of Samsung Medical Center, Korea. HuLa-PC, a cell line derived from posterior commissure of the larynx, was obtained from ATCC (Gaithersburg, Maryland) and cultured in Dermal Cell Basal Medium (ATCC^®^ PCS-200-030TM).

### qRT-PCR

Total RNA was isolated from tissues and cell lines with TRIzol reagent (Invitrogen, Thermo Fisher Scientific), and then reverse transcribed using Evo M-MLV RT Kit with gDNA Clean for qPCR (AG11711). qRT-PCR was conducted using SYBR Green Premix Pro Taq HS qPCR Kit (AG11701) for mRNA with ABI 7500 Real-Time PCR System (Life Technologies, Shanghai, China), and the housekeeping gene GAPDH was used as an internal control. The primers were synthesized by Sangon Biotech (Shanghai) Co., Ltd. The sequences of all primers are listed in Additional file 9: Table S3.

### Immunofluorescence of tissue microarrays (TMAs)

The TMAs, including 72 paired patients with head and neck squamous cell carcinoma (HNSCC), were obtained from Eye and ENT Hospital, Fudan University (FDEENT). After filtering out patients with hypopharyngeal carcinoma and fragments, 51 patients with LSCC remained. Immunofluorescence staining was performed on 51 paired samples to detect GPT (ab236658, ABCAM, 1:100 dilution), SMS (ab247063, ABCAM, 1:3000 dilution), and DAPI (G1012, Servicebio) expression. TMAs were scanned using the microscope slide scanner (Pannoramic MIDI: 3Dhistech) after staining with the appropriate antibodies. Antibodies against GPT (ab236658) and SMS (ab247063) were purchased from Abcam (Cambridge, MA, USA). All scanned cores or slides were individually detected and quantified using Image J software [36]. GPT and SMS positive staining was calculated relative to DAPI staining. After integrating survival information, we conducted receiver operating characteristic (ROC) curve and survival analysis of risk score (GPT + SMS). ROC analysis was performed to identify the sensitivity and specificity of risk score and the optimal cutoff value for predicting overall survival (OS). According to the cutoff value, patients were divided into two groups. The prognostic difference between the two groups was then analyzed using a Kaplan–Meier estimator with a log rank test.

### Independent prognosis analysis

In order to further evaluate the predictive ability of the prognostic signature, we used Cox regression analysis to analyze whether the prognosis signature could act as an independent prognostic factor for LSCC in both TCGA cohort and FDEENT cohort.

### Building a nomogram based on the prognostic signature

Based on results of independent prognosis analysis, we built a nomogram by “rms” package to facilitate the application of the prognosis signature. Calibration curves and ROC curves were used to verify the validity of the signature.

### Statistical analysis

All statistical analyses were carried out by using R software (version: × 64 3.6.1) and GraphPad Prism 7 software. A *p*-value < 0.05 was regarded as statistically significant. Differential expression analysis of MRGs were performed by Wilcox test. The survival analysis was completed using the log-rank test with a threshold of *p*-value < 0.05. Independent prognosis analysis was done by univariate and multivariate Cox regression analysis.

## Results

### Identification of differentially expressed MRGs

**Fig. 1:**
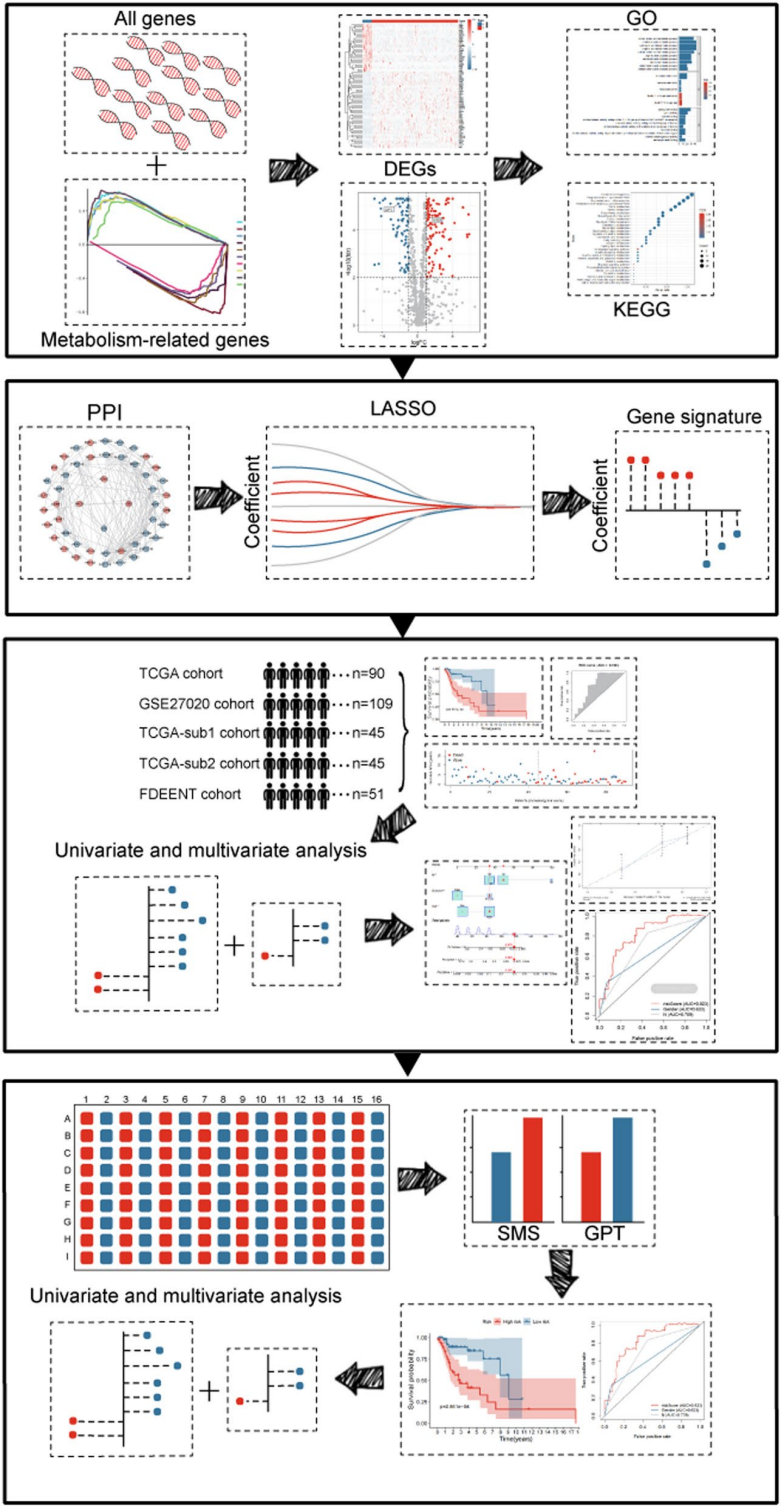
The flow chart of this study

**Fig. 2:**
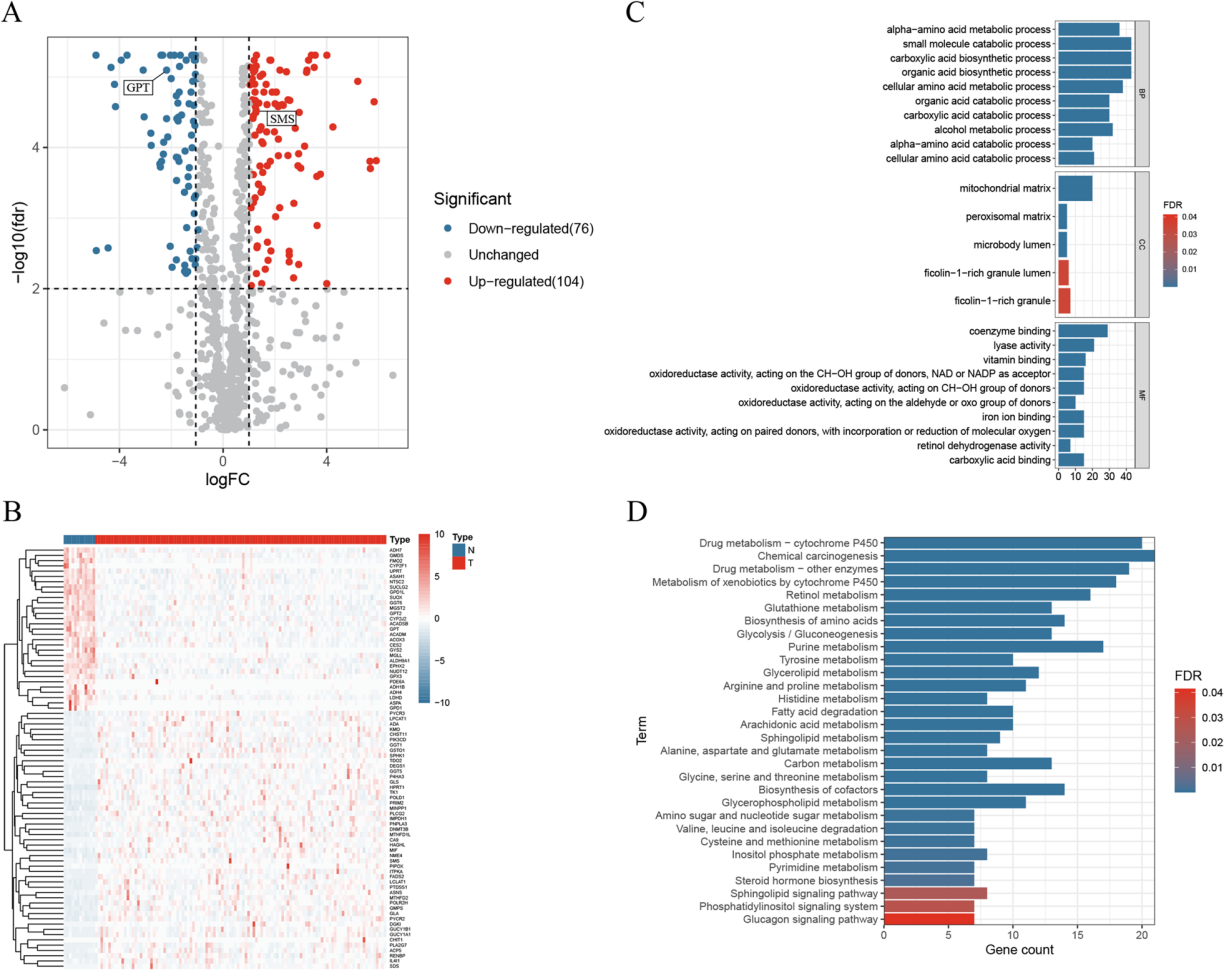
Differentially expressed MRGs and corresponding pathway enrichment. **A** Volcano plot of 180 MRGs, red indicating up-regulated MRGs and blue indicating down-regulated MRGs. **B** Heatmap of the top 80 MRGs with significant adjusted *p*-value. **C** GO terms with significant difference. **D** KEGG pathways with significant difference

The flow chart of this study is presented in Fig. 1. We obtained the RNA-seq data of MRGs from TCGA and GSEA, and 180 differentially expressed MRGs were screened out by the filter conditions (Additional file 10: Table S4). The differentially expressed MRGs were visualized in volcano plot (Fig. 2A) and the top 80 MRGs with significant adjusted *p*-value were shown in a heatmap (Fig. 2B).

### Pathway enrichment analysis and PPI analysis

**Fig. 3:**
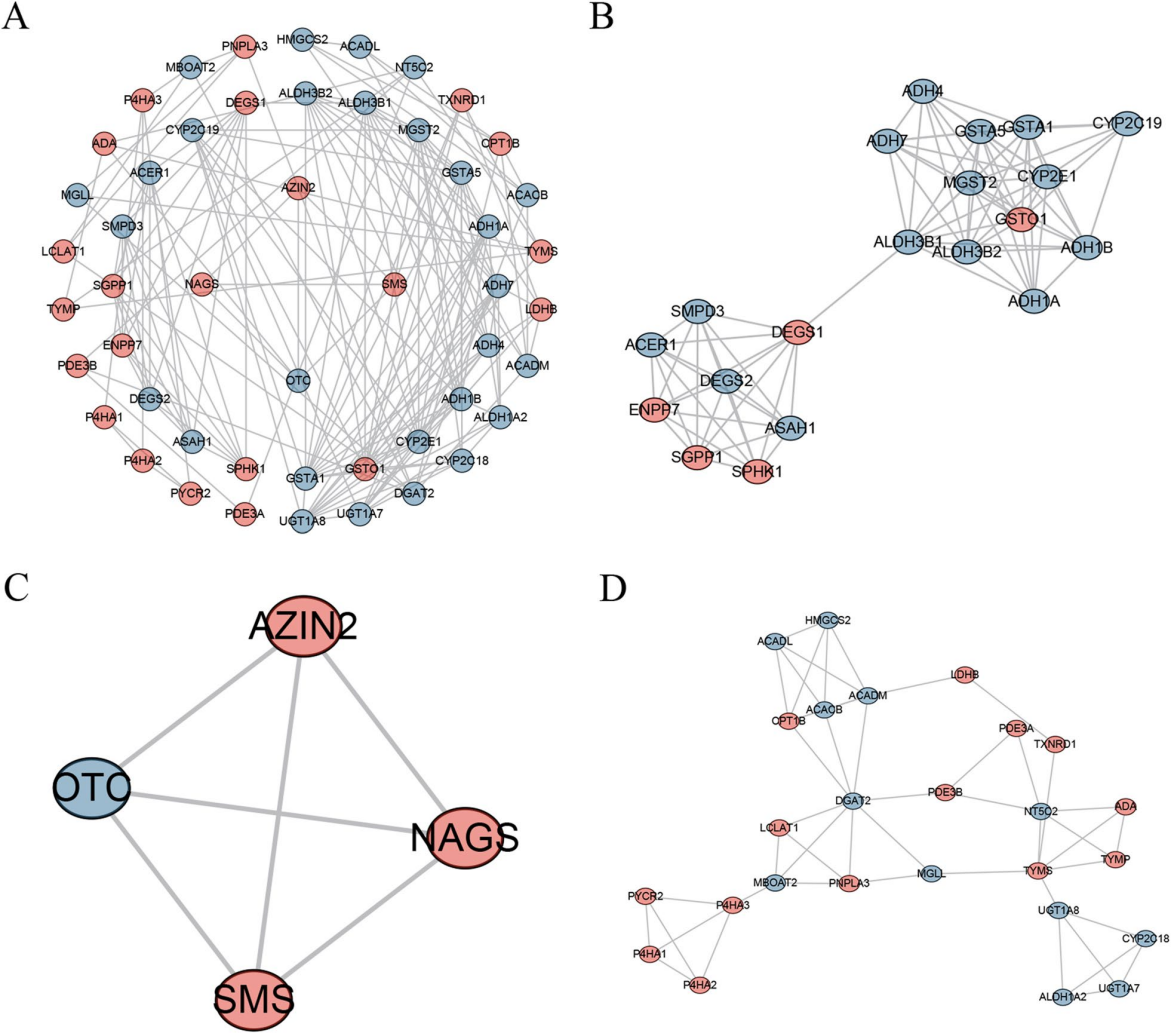
PPI analysis of different expressed MRGs. **A** Visualization of the PPI network, red indicating up-regulated MRGs and blue indicating down-regulated MRGs. **B**, **D** Three most important sub-networks

The results of GO analysis showed that the differentially expressed MRGs were mainly enriched in small molecule catabolic process, carboxylic acid biosynthetic process, organic acid biosynthetic process, cellular amino acid metabolic process, alpha-amino acid metabolic process, alcohol metabolic process, organic acid catabolic process, carboxylic acid catabolic process, coenzyme binding, and fatty acid metabolic process (Fig. 2C). KEGG analysis demonstrated that the differentially expressed MRGs were significantly accumulated in Chemical carcinogenesis, Drug metabolism—cytochrome P450, Drug metabolism—other enzymes, Metabolism of xenobiotics by cytochrome P450, Purine metabolism, Retinol metabolism, Biosynthesis of amino acids, Biosynthesis of cofactors, Glutathione metabolism, Glycolysis / Gluconeogenesis (Fig. 2D). At the same time, 173 MRGs with high connectivity were used to establish a PPI network (Additional file 5: Fig. S5). Three most important sub-networks were screened out (Fig. 3A–D). The enrichment results of sub-networks are presented in Additional file 11: Table S5. The results of pathway enrichment analysis showed that hub MRGs of subnetwork1 were mainly enriched in drug metabolism of cytochrome P450, sphingolipid metabolism, tyrosine metabolism glycolysis, fatty acid degradation and glutathione metabolism; hub MRGs of subnetwork2 were mainly enriched in arginine and proline metabolism, biosynthesis of amino acids, 2-oxocarboxylic acid metabolism, cysteine and methionine metabolism and glutathione metabolism; hub MRGs of subnetwork3 were mainly enriched in glycerolipid metabolism, arginine and proline metabolism, retinol metabolism, PPAR signaling pathway, fatty acid degradation, glucagon signaling pathway, purine metabolism, pyrimidine metabolism, fatty acid metabolism and drug metabolism. These results indicated that the clustering of hub modules was mainly on metabolic functions and metabolic pathways. Moreover, the results verified that the MRGs that we identified were highly correlated with metabolic functions and metabolic pathways.

### Construction and validation of a prognostic signature based on MRGs

**Fig. 4:**
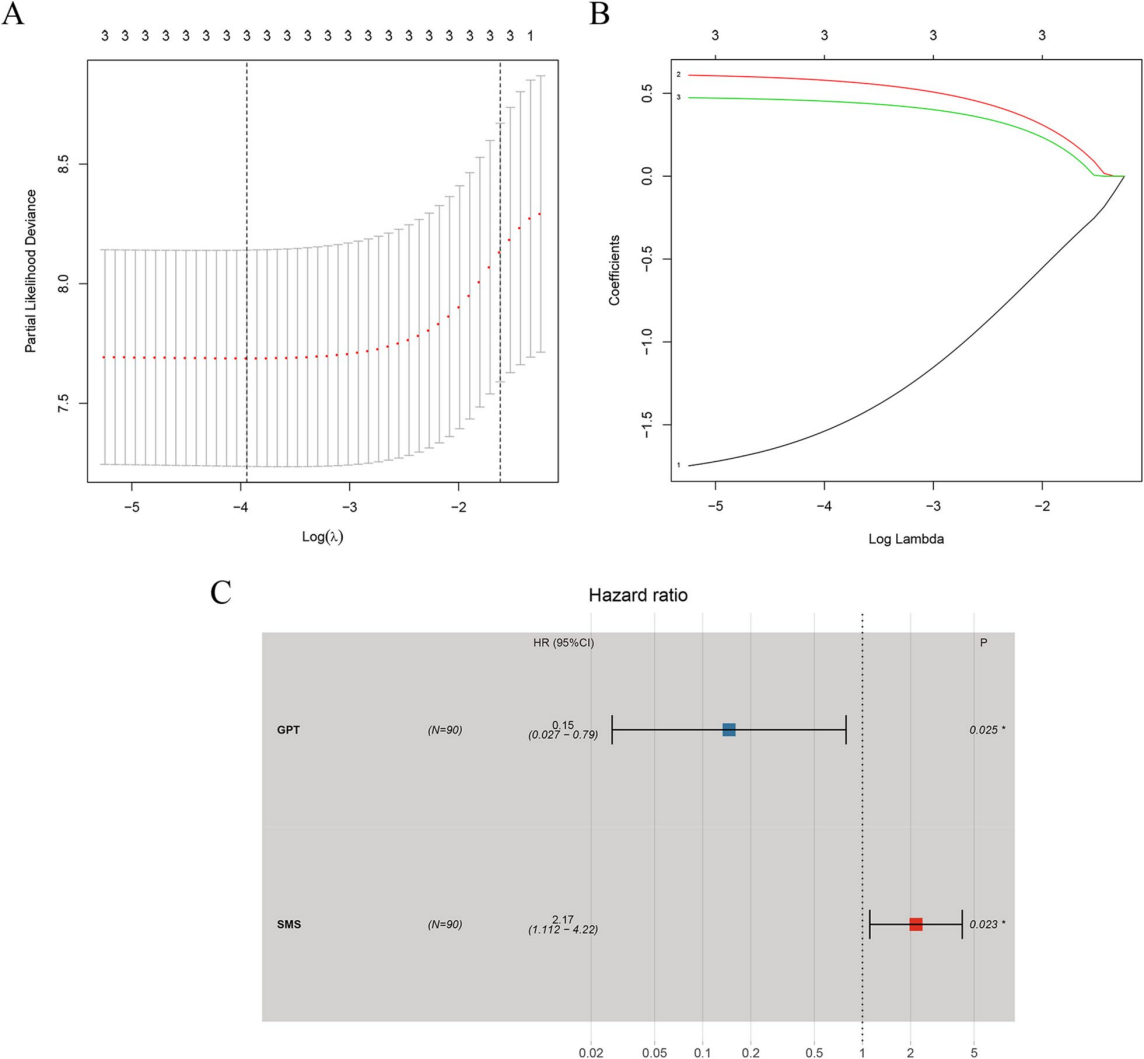
Building of the prognostic signature. **A** LASSO deviance profiles. **B** LASSO coefficient profiles. **C** Multivariate Cox regression analysis

In TCGA cohort, 14 prognostic genes were identified via univariate Cox regression analysis (Table 1). Subsequently, an MRGs-related prognostic signature was constructed by LASSO analysis (Fig. 4) and multivariate Cox regression analysis (Fig. 4C, Table 2). The risk score of the prognostic signature could be calculated as follows: (Exp GPT * − 1.922494852) + (Exp SMS * 0.772558166).

**Table 1:** Univariate Cox regression analysis for the identification of prognosis-related MRGs

id	HR	HR.95L	HR.95H	*p*-value
SMS	2.735130093	1.415449884	5.285200633	0.002755758
PCYT1B	2.605329136	1.344789285	5.04743753	0.004540788
GPT	0.12408443	0.025648695	0.600301342	0.009474802
MGLL	2.051909647	1.17920387	3.570487943	0.010983847
PSPH	1.614292836	1.111175448	2.345211427	0.011964648
MBOAT2	1.79778777	1.124607055	2.873929031	0.014262082
FMO2	1.753322337	1.108047542	2.774374838	0.016477913
POLD1	0.274715851	0.09436419	0.799761	0.017797792
CES2	1.864178468	1.088970307	3.191236104	0.023164597
ALDH1A2	10.29092415	1.355280595	78.14110257	0.024203614
HPRT1	2.718622623	1.093636296	6.758105039	0.031348818
ACP5	0.56716861	0.33362033	0.964210521	0.036208727
FTH1	2.408171746	1.01888244	5.691815787	0.045221492
ACPP	2.242975417	1.005323978	5.004295956	0.048503256

**Table 2:** Multivariate Cox regression analysis for the identification of prognosis-related MRGs

id	coef	HR	HR.95L	HR.95H	*p*-value
GPT	− 1.922494852	0.146241655	0.027088482	0.789509792	0.025437938
SMS	0.772558166	2.165298368	1.111887633	4.216718384	0.023095538

**Fig. 5:**
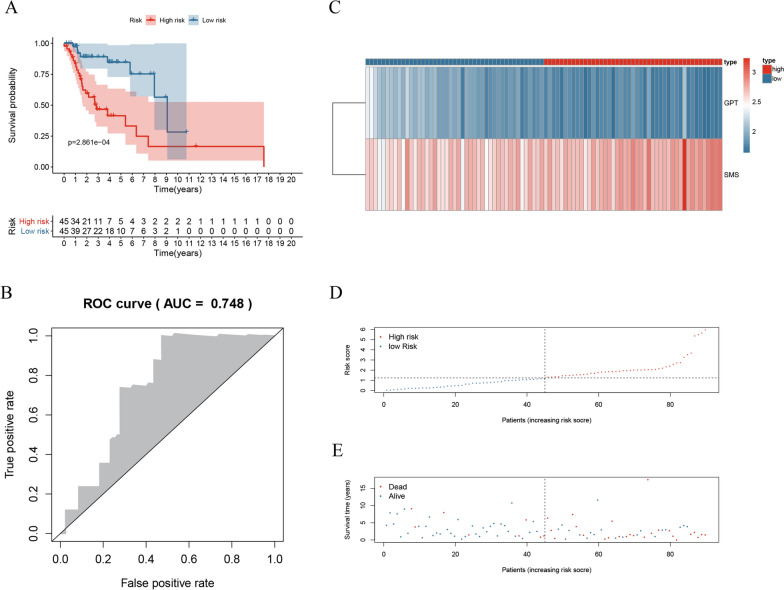
Internal validation of the prognostic signature in TCGA cohort. **A** KM survival analysis of high-risk and low-risk patients. **B** ROC curve of TCGA cohort. **C** Heatmap of GPT and SMS expression. **D**, **E** Survival status and risk score of patients

To evaluate the predictive effectiveness of the prognostic signature, we divided the patients of the TCGA cohort (n = 90) into the high- and low-risk groups according to the median risk score. The overall survival (OS) of the high-risk group was poorer than that of the low-risk group (Fig. 5A). The area under curves (AUC) of TCGA cohort was 0.748 (Fig. 5B). The survival status, risk score and the hub MRGs expression of the two groups are displayed in Fig. 5C–E.

In the external validation (GSE27020 cohort, n = 109), the DFS of the high-risk group was poorer than that of the low-risk group (Additional file 1: Fig. S1). The DFS status, risk score and the hub MRGs expression of the two groups are displayed in Additional file 1: Fig. S1. The results suggested that the prognostic signature we developed was effective not only for stratification of laryngeal cancer survival, but also for recurrence patients with LSCC.

In the internal validation (TCGA-sub1 cohort, n = 45; TCGA-sub2 cohort, n = 45), the OS of the high-risk group was poorer than that of the low-risk group in both cohorts (Additional file 2: Fig. S2 and Additional file 3: Fig. S3). The AUCs of both cohorts are shown in Additional file 2: Fig. S2 and Additional file 3: Fig. S3.

### Higher SMS expression and lower GPT expression in LSCC tissues indicating a poorer outcome

**Fig. 6:**
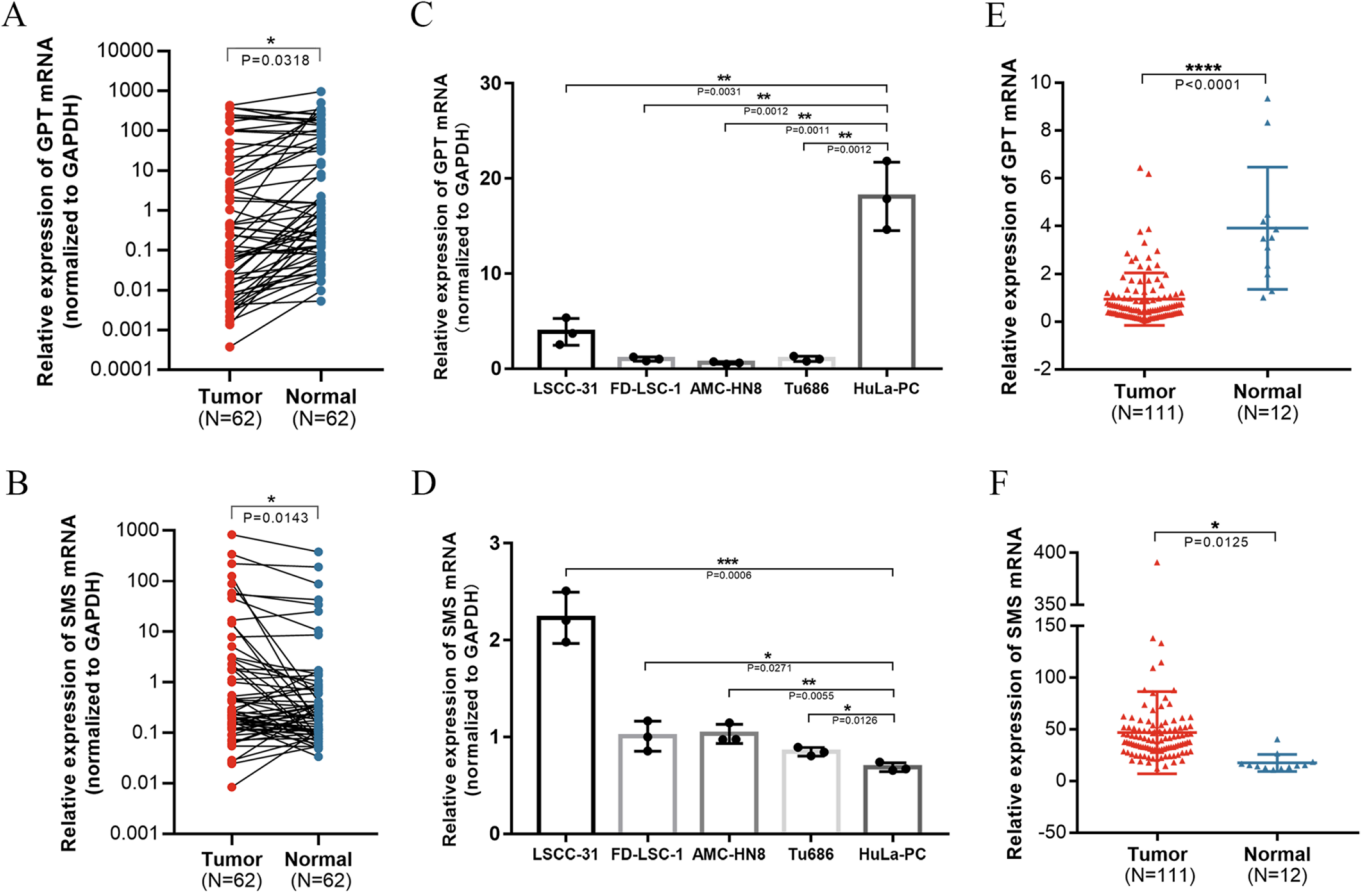
SMS and GPT expression in LSCC patients and cell lines. **A** GPT is down-regulated in LSCC tissues compared to adjacent normal tissues. **B** SMS is up-regulated in LSCC tissues compared to adjacent normal tissues. **C** GPT is down-regulated in LSCC cell lines compared to HuLa-PC, a cell line derived from posterior commissure of the larynx. **D** SMS is up-regulated in LSCC cell lines compared to HuLa-PC. **E** Relative expression of GPT in TCGA. **F** Relative expression of SMS in TCGA

**Fig. 7:**
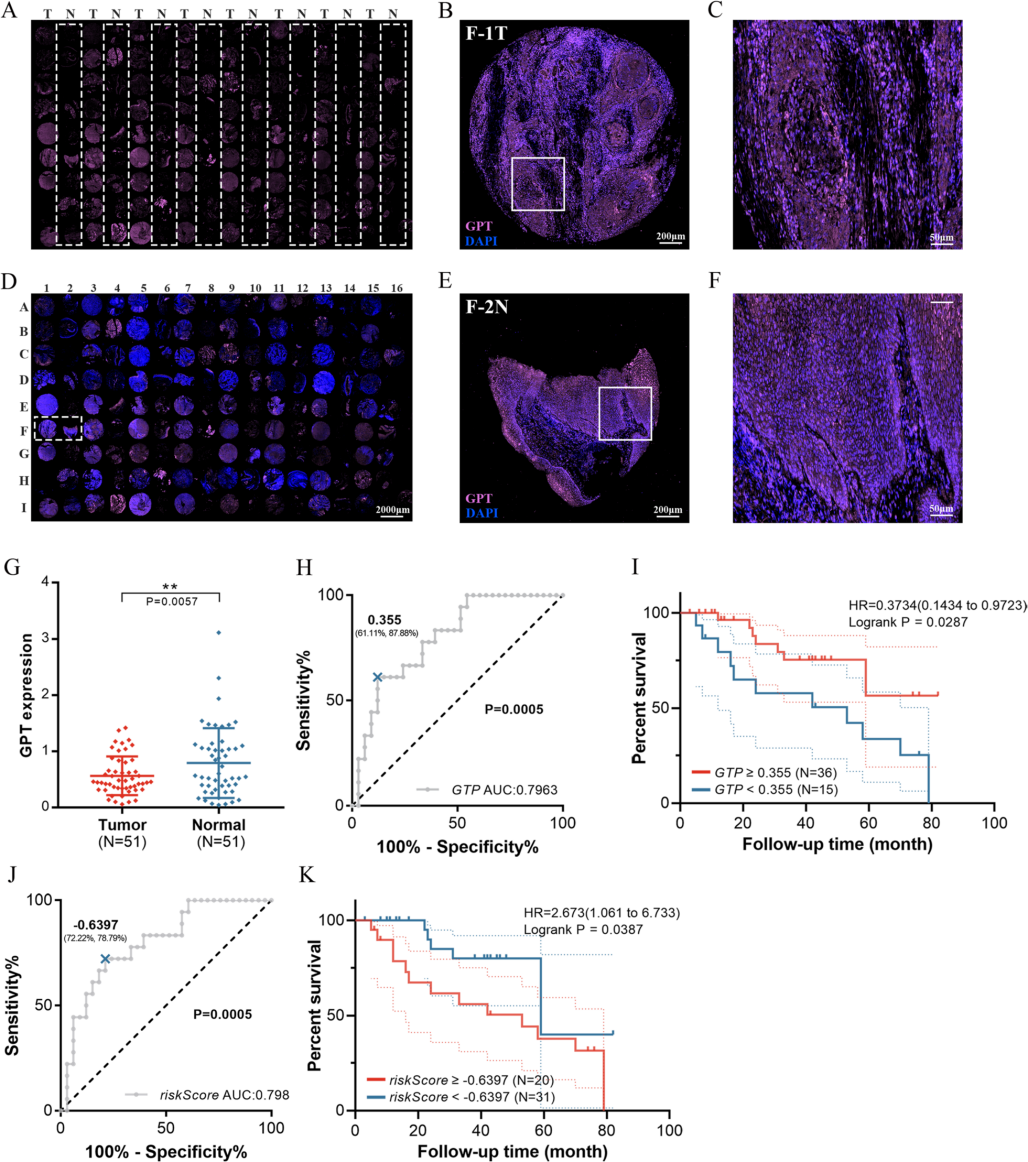
External validation of the prognosis signature in FDEENT cohort. **A** GPT immunofluorescence staining on TMA. **B**, **C** Merged immunofluorescence staining on F-1 T. **D** Overview of merged immunofluorescence staining on TMA. **E**, **F** Merged immunofluorescence staining on F-2 N. **G** Quantification of the immunofluorescence signals of GPT. **H** ROC curve of GPT prediction. **I** Survival analysis of GPT by the KM plotter in LSCC. **J** ROC curve of risk score (GPT + SMS) prediction. **K** Survival analysis of risk score (GPT + SMS) by the KM plotter in LSCC

In order to verify the expressions of GPT and SMS, 62 LSCC tissues with their paired adjacent normal tissues and five cell lines were selected and tested by qRT-PCR. The LSCC tissues showed up-regulated SMS compared to the adjacent normal tissues, while GPT was opposite (Fig. 6A, B), which was in line with our findings from TCGA database (Fig. 6E, F). Consistent results were also observed in cell lines (Fig. 6C, D). In TMA (FDEENT cohort, n = 51), we noted that GPT protein (in pink) was located primarily in the epithelium (Fig. 7A–F) and down-regulated in LSCC tissues compared to adjacent normal tissues (Fig. 7G). ROC curve showed that the AUC was 0.7963 (*p* = 0.0005) and a value of 0.355 was the best balance between the sensitivity and specificity for predicting OS (Fig. 7H). Survival analysis showed that low GPT expression was related to a lower OS rate among LSCC patients (*p* = 0.0287) (Fig. 7I). SMS protein was analyzed in the same way and the results are displayed in Additional file 4: Fig. S4.

To validate our findings, we integrated GPT and SMS expression according to the prognostic signature (GPT * − 1.922494852 + SMS * 0.772558166). In FDEENT cohort (n = 51), we found that the AUC of risk score (GPT + SMS) was 0.798 (*p* = 0.0005) (Fig. 7J) and the OS of the high-risk group was poorer than that of the low-risk group (*p* = 0.0387) (Fig. 7K). These results were also consistent with our findings.

### Independent prognosis analysis

**Fig. 8:**
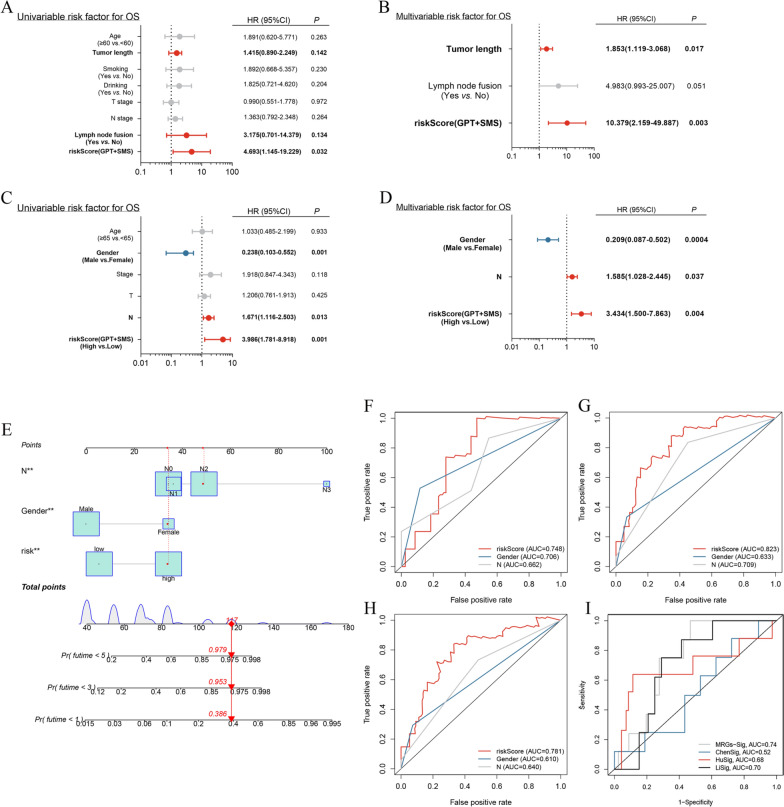
Independent prognostic analysis and construction of a nomogram based on independent prognostic factors for predicting OS. **A**, **B** Results of univariate and multivariate Cox regression analysis of FDEENT cohort. **C**, **D** Results of univariate and multivariate Cox regression analysis of TCGA cohort. **E** Nomogram based on the independent prognostic factors of TCGA cohort. **F**–**H** ROC curve of independent prognostic factors in 1-, 3-, and 5-year OS. **I** Comparison of prognostic signature with other models

The FDEENT cohort and TCGA cohort were assessed by independent prognosis analysis. The results showed that the risk score and tumor length were independent prognostic factors in FDEENT cohort (Fig. 8A, B), while the risk score, gender and stage N were independent prognostic factors in TCGA cohort (Fig. 8C, D).

### Building a nomogram based on the prognostic signature

Using the TCGA cohort, we built a nomogram (Fig. 8E) to make the prognostic signature more convenient for clinical application. Based on the score of subitems (including the risk score and other significant independent risk factors), the 1-, 3-, and 5-year OS of patients with LSCC could be predicted. Calibration curves indicated that the predicted value of the risk score was closer to the actual observation value with the increase in number of years (Additional file 5: Fig. S5). At the same time, we found that the AUC of the risk score was higher than that of the other significant independent risk factors in 1-, 3-, and 5-year OS (Fig. 8F–H).

## Discussion

LSCC, which is of epithelial origin, is a most common tumor of the upper respiratory tract [37]. Poor living habits, such as smoking and alcohol consumption and imbalanced diet, are the main risk factors contributing to laryngeal cancer [38]. As an important feature of tumors, metabolic abnormality could adjust to the microenvironment to meet the requirement of the constantly growing tumor cells [39]. Recently, more and more studies have focused on tumor metabolism to elucidate the pathogenesis of LSCC. For example, Hu reported that PCK2 down-regulation inhibited the invasion, migration, and proliferation of laryngeal cancer under hypoxia, and therefore could be used as a new strategy for laryngeal cancer therapy [40]. Using liquid chromatograph-mass spectrometry (LC–MS) technology and molecular biology experiments, Zhao et al. found that fatty acid desaturase 1 (FADS1) promoted the progression of LSCC through activating AKT/mTOR signaling pathway [41].

In this study, we utilized several public databases and screened out prognostic metabolic markers of LSCC by bioinformatics analysis and molecular biology experiment. First, differential expression analysis was conducted and 180 differentially expressed MRGs were screened out. We noticed that the results of GO and KEGG pathway enrichment analyses were all related to metabolism, such as purine metabolism, glycometabolism, lipid metabolism, and pyrimidine metabolism. Then, using the univariate Cox regression analysis, we found 14 MRGs significantly related to OS of LSCC patients (*p* < 0.01, Table 1). To avoid overfitting the prognostic risk signature, we performed least absolute shrinkage and selection operator (LASSO) analysis on TCGA cohort to further shrink the scope of gene screening [42], and the optimal values of the penalty parameter were determined by ten-fold cross-validation (Fig. 4B). Finally, by multivariate Cox regression analysis, two MRGs (GPT and SMS) conforming to the proportional hazards assumption were selected to build a prognostic signature.

Cellular metabolism plays a crucial role in tumor progression, and metabolic reprogramming is one of the hallmarks of tumor [43]. Cancer cells proliferate rapidly due to their special metabolic characteristics [44]. For example, gluconeogenesis, a crucial phenotype for glucose homeostasis of tumor cells, indicates that cells obtain energy from malnourished environment to sustain their rapid proliferation by using non-carbohydrate carbon substrates to generate pyruvate [11]. Glutamic pyruvic transaminase (GPT), also known as alanine aminotransferase (ALT), participates in the intermediary metabolism of glucose and amino acids, catalyzing the reversible transamination between alanine and 2-oxoglutarate to generate pyruvate and glutamate [12]. There are two GPT subtypes in mammals [19]. The role of GTP2 in cancer has been reported in several studies [13–18]. PIK3CA mutations reprogram glutamine metabolism by upregulating GPT2 in colorectal cancer cells. Also, aminooxyacetate (AOA) suppresses tumor proliferation of colorectal cancer with PIK3CA mutations by inhibiting enzymatic activity of GPT2 [14]. Moreover, GPT2 promotes tumorigenesis of breast cancer cells by activating sonic hedgehog signaling [13]. GPT1 is generally considered to be a biomarker in liver diseases. Several studies have found a relationship between an elevated aminotransaminase (AST)/alanine aminotransaminase (ALT) ratio and a poorer prognosis in patients with hepatocellular [45], bladder [46], testicular [47], prostate [48], pancreatic carcinomas [49] and head and neck cancer [50–53]. These results indicate that the decrease of GPT1 expression in serum is related to the poor prognosis of oncology patients. Similarly, in this study, we found that lower GPT1 expression in LSCC tissues could indicate a poorer outcome. However, few studies have been performed on the regulatory role of GPT1 in cancer. Whether GPT1 has the similar function in regulating energy metabolism and tumor proliferation like GPT2 needs in-depth investigation.

In addition to the metabolism of glucose and amino acids, polyamine metabolism is also critical in tumorigenesis [54–56]. Spermine synthase (SMS), a highly specific aminopropyltransferase [57], is a biosynthetic enzyme [58]. SMS overexpression can upregulate polyamine level, which maintains the growth and proliferation of tumor cells [54]. Moreover, SMS can block Bim transcription by reducing spermidine-mediated inhibition of FOXO3a acetylation, to maintain tumor growth [22]. In plasma of patients with triple-negative breast cancer, high mRNA expression of SMS is highly related to metastasis and poor prognosis [59], which is similar to our results. In our study, we found that SMS transcript level was up-regulated in LSCC tissues through RNA-seq data (Fig. 6F). Subsequently, we verified and observed the same SMS transcript level changes through qRT-PCR (Fig. 6B). In order to further explore the protein level of SMS, we conducted the immunofluorescence staining assay. Contrary to transcript level, SMS protein level was significantly down-regulated in LSCC tissues (Additional file 4: Fig. S4). Interestingly, SMS protein expression was not associated with the prognosis of LSCC patients, but the combined signature based on SMS and GPT was associated with the prognosis of LSCC patients. For the inconsistency between SMS transcriptional level and protein level, we considered the following possibilities: First, it is likely that there are other proteins or genes involved in the regulation of SMS expression, including interacting proteins, miRNAs, lncRNAs and circRNAs. Second, this is possibly caused by different post-transcriptional modifications (i.e., polyadenylation of the 3′ end) and post-translational modification (i.e., acetylation, methylation, ubiquitination and phosphorylation). Finally, high-throughput RNA sequencing technique (RNA-seq), also named transcriptome sequencing technology, can only reflect the transcript level of genes. Therefore, it is reasonable and scientific to verify the RNA-seq results by qRT-PCR, rather than Western blot and immunofluorescence staining. We hope that these results may shed light on the relationship between spermine metabolism and LSCC.

Concordant with previous reports, SMS was up-regulated while GPT was down-regulated in LSCC tissues compared to adjacent normal tissues as verified by clinical samples and multiple datasets in our study. Based on the expressions of SMS and GPT, LSCC patients were divided into the high- and low-risk groups. Five different cohorts, including internal and external validation cohorts, were analyzed and tested to verify the accuracy and reliability of the prognostic signature. Based on the clinical information and the risk score, we performed independent prognostic analysis in TCGA cohort (n = 90). The results showed that the risk score, gender, and stage N were independent prognostic factors in TCGA cohort. In order to demonstrate the robustness of the prognostic signature, independent prognostic analysis was also performed in another independent cohort (FDEENT cohort). Again, the results revealed that the risk score was an independent prognostic factor of LSCC patients in FDEENT cohort. Although the results produced by different cohorts and clinical information were not exactly the same, the retrospective different cohorts suggested that the prognostic signature was reliable and independent and could classify the LSCC patients into two groups with different OS outcomes. Moreover, we performed Kaplan–Meier survival analysis based on the stratification of clinicopathological factors. As shown in Additional file 6: Fig. S6, the prognosis of high-risk group was significantly worse than that of low-risk group in almost all subgroups, suggesting the effectiveness and universality of the signature. Therefore, a nomogram based on the risk score was developed, which might contribute to the decision making of LSCC treatment.

In the previous studies, Chen [60] and Li [61] developed metabolism-related signatures to predict the prognosis of head and neck squamous cell carcinoma, while Hu [62] identified a combined lipid metabolism-related signature for oral squamous cell carcinoma (OSCC). To our knowledge, our study is the first to develop and validate an MRG signature for LSCC. This signature showed a higher AUC value than those by other studies based on TCGA cohort (Fig. 8I), indicating a higher sensitivity and specificity. In addition, compared with other prognostic signatures composed of multiple genes, this signature derived from two MRGs is easier to apply in clinical practice.

There are some deficiencies in our research. First, only five cohorts were incorporated to verify this prognostic signature. In future study, more cohorts and LSCC patients should be included to reduce the deviation of racial and geographic distribution. Second, our findings were based on retrospective studies, and prospective clinical trials should be conducted. More importantly, in vivo and in vitro experiments on GPT and SMS are needed.

## Conclusions

In summary, our study identified differentially expressed MRGs and constructed a novel prognostic signature derived from MRGs in LSCC for the first time. Being able to distinguish LSCC patients with different risk scores, this novel signature could make precise prognosis of LSCC. A nomogram based on this prognostic signature was also developed, which may help in the clinical treatment of LSCC.

## Supplementary Information

**Additional file 1: Figure S1.** External validation of the prognostic signature in GSE27020 cohort. **A** KM survival analysis of high-risk and low-risk patients. **B** ROC curve of GSE27020 cohort. **C** Heatmap of GPT and SMS expression. **D**, **E** Survival status and risk score of patients.

**Additional file 2: Figure S2.** Internal validation of the prognostic signature in TCGA-sub1 cohort. **A** KM survival analysis of high-risk and low-risk patients. **B** ROC curve of TCGA-sub1 cohort. **C** Heatmap of GPT and SMS expression. **D**, **E** Survival status and risk score of patients.

**Additional file 3: Figure S3.** Internal validation of the prognostic signature in TCGA-sub2 cohort. **A** KM survival analysis of high-risk and low-risk patients. **B** ROC curve of TCGA-sub2 cohort. **C** Heatmap of GPT and SMS expression. **D**, **E** Survival status and risk score of patients.

**Additional file 4: Figure S4.** External validation of SMS expression and prognosis in FDEENT cohort. **A** SMS immunofluorescence staining on TMA. **B**, **C** Merged immunofluorescence staining on F-1 T. **D** Overview of merged immunofluorescence staining on TMA. **E**, **F** Merged immunofluorescence staining on F-2 N. **G** Quantification of the immunofluorescence signals of SMS. **H** ROC curve of SMS prediction. **I** Survival analysis of SMS by the KM plotter in LSCC.

**Additional file 5: Figure S5.** PPI analysis and verification of the nomogram. **A**, **B** Visualization of the PPI network. **C**, **E** Calibration curve of prognostic signature for 1-, 3-, and 5-year OS.

**Additional file 6: Figure S6.** Kaplan–Meier survival analysis for all LSCC patients according to the prognostic signature stratified by clinicopathological risk factors. **A**, **B** The Kaplan–Meier stratified by age. **C**, **D** The Kaplan–Meier stratified by gender. **E**, **F** The Kaplan–Meier stratified by stage N. **G**, **H** The Kaplan–Meier stratified by stage. **I**, **J** The Kaplan–Meier stratified by stage T.

**Additional file 7: Table S1.** Clinical information of TCGA cohort.

**Additional file 8: Table S2.** Metabolic-related pathways and corresponding genes from GSEA.

**Additional file 9: Table S3.** Sequences of all primers used in this study.

**Additional file 10: Table S4.** List of differentially expressed MRGs.

**Additional file 11: Table S5.** Enrichment analysis of sub-networks.

## Data Availability

The datasets analyzed during the current study are available from the corresponding author Liang Zhou on reasonable request. The data that support the findings of this study are available at the TCGA data portal (https://tcga-data.nci.nih.gov/tcga/) and the comprehensive Gene Expression Omnibus (GEO; https://www.ncbi.nlm.nih.gov/geo/).

## Abbreviations

LSCC: Laryngeal squamous cell carcinoma
MRGs: Metabolism-related genes
TCGA: The Cancer Genome Atlas
GSEA: Gene set enrichment analysis
PPI: Protein–protein interaction
LASSO: Least absolute shrinkage and selection operator
ROC: Receiver operating characteristic
qRT-PCR: Quantitative reverse transcriptase PCR
TMA: Tissue microarray
GEO: Gene Expression Omnibus
FC: Fold change
GO: Gene ontology
KEGG: Kyoto Encyclopedia of Genes and Genomes
DFS: Disease-free survival
HNSCC: Head and neck squamous cell carcinoma
FDEENT: Eye and ENT Hospital, Fudan University
OS: Overall survival
FADS1: Fatty acid desaturase 1
GPT: Glutamic pyruvic transaminase
SMS: Spermine synthase
SRS: Snyder-Robinson syndrome
OSCC: Oral squamous cell carcinoma
