# Supplementary figures and images for: A novel signature derived from metabolism-related genes GPT and SMS to predict prognosis of laryngeal squamous cell carcinoma
Source: Cancer Cell Int. 2022 Jul 8;22:226. doi: 10.1186/s12935-022-02647-2 (PMC9270735; doi:10.1186/s12935-022-02647-2)

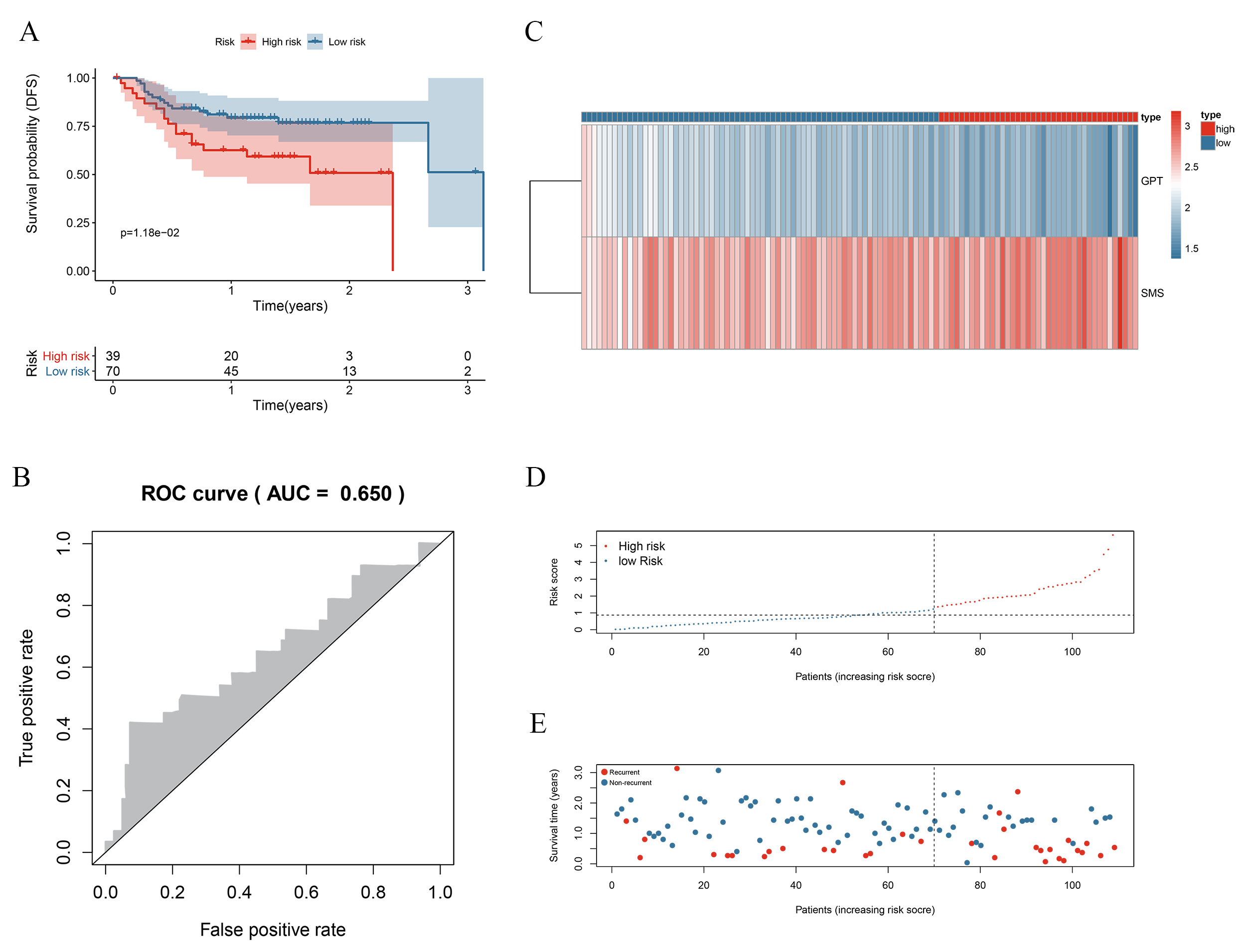

Supplement: Supplementary file 1 — Additional file 1: Figure S1. External validation of the prognostic signature in GSE27020 cohort. A KM survival analysis of high-risk and low-risk patients. B ROC curve of GSE27020 cohort. C Heatmap of GPT and SMS expression. D, E Survival status and risk score of patients. [file 12935_2022_2647_MOESM1_ESM.tif]

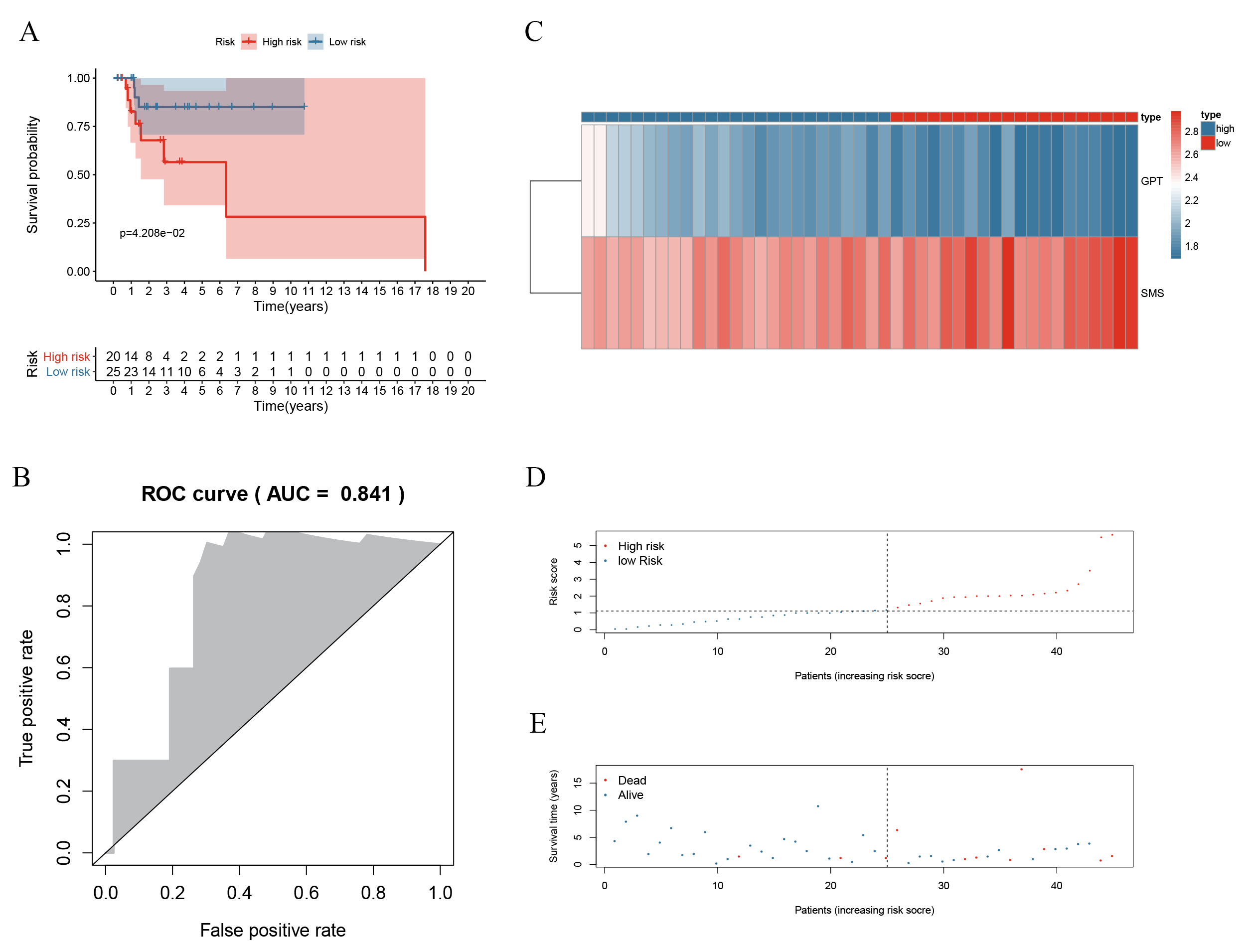

Supplement: Supplementary file 2 — Additional file 2: Figure S2. Internal validation of the prognostic signature in TCGA-sub1 cohort. A KM survival analysis of high-risk and low-risk patients. B ROC curve of TCGA-sub1 cohort. C Heatmap of GPT and SMS expression. D, E Survival status and risk score of patients. [file 12935_2022_2647_MOESM2_ESM.tif]

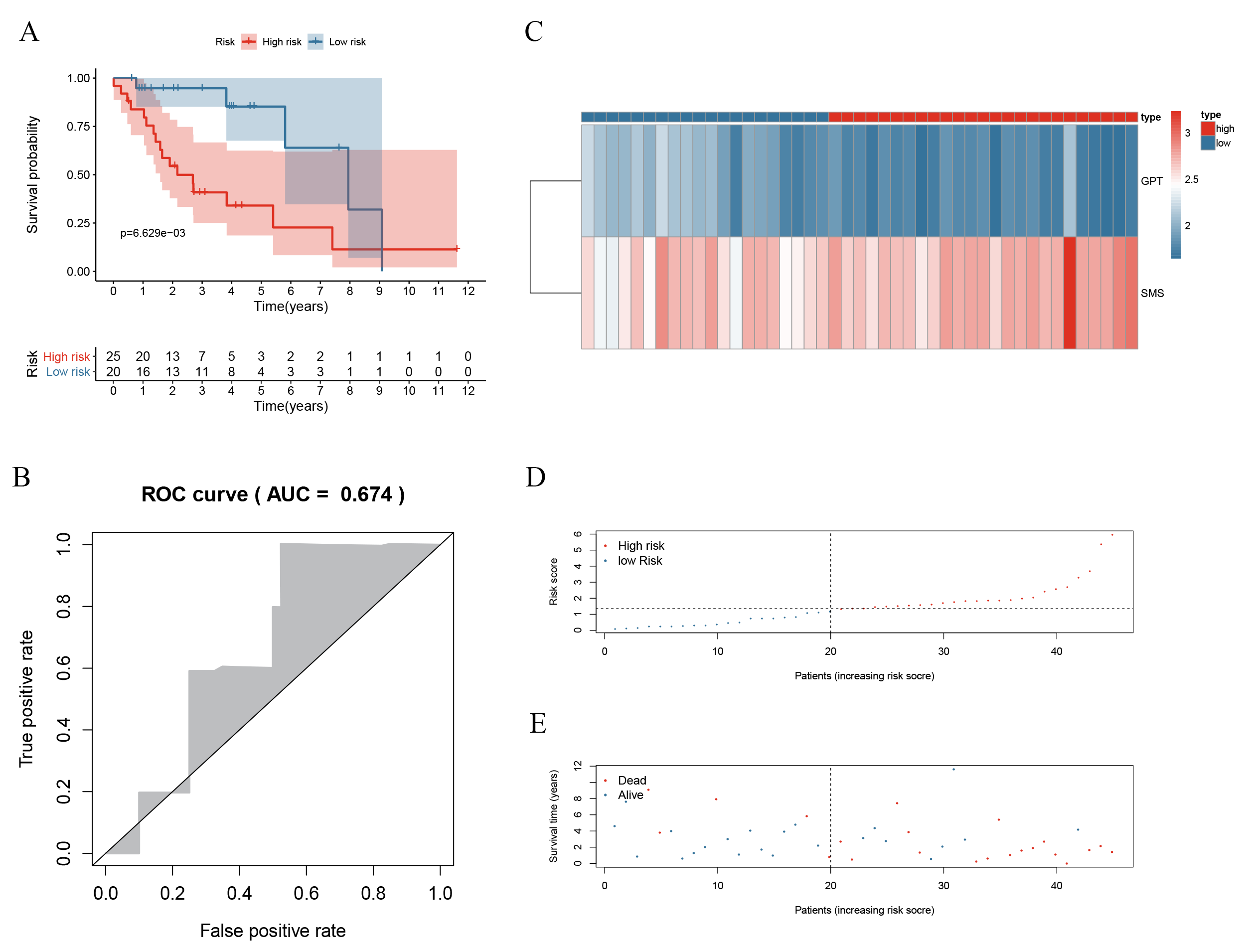

Supplement: Supplementary file 3 — Additional file 3: Figure S3. Internal validation of the prognostic signature in TCGA-sub2 cohort. A KM survival analysis of high-risk and low-risk patients. B ROC curve of TCGA-sub2 cohort. C Heatmap of GPT and SMS expression. D, E Survival status and risk score of patients. [file 12935_2022_2647_MOESM3_ESM.tif]

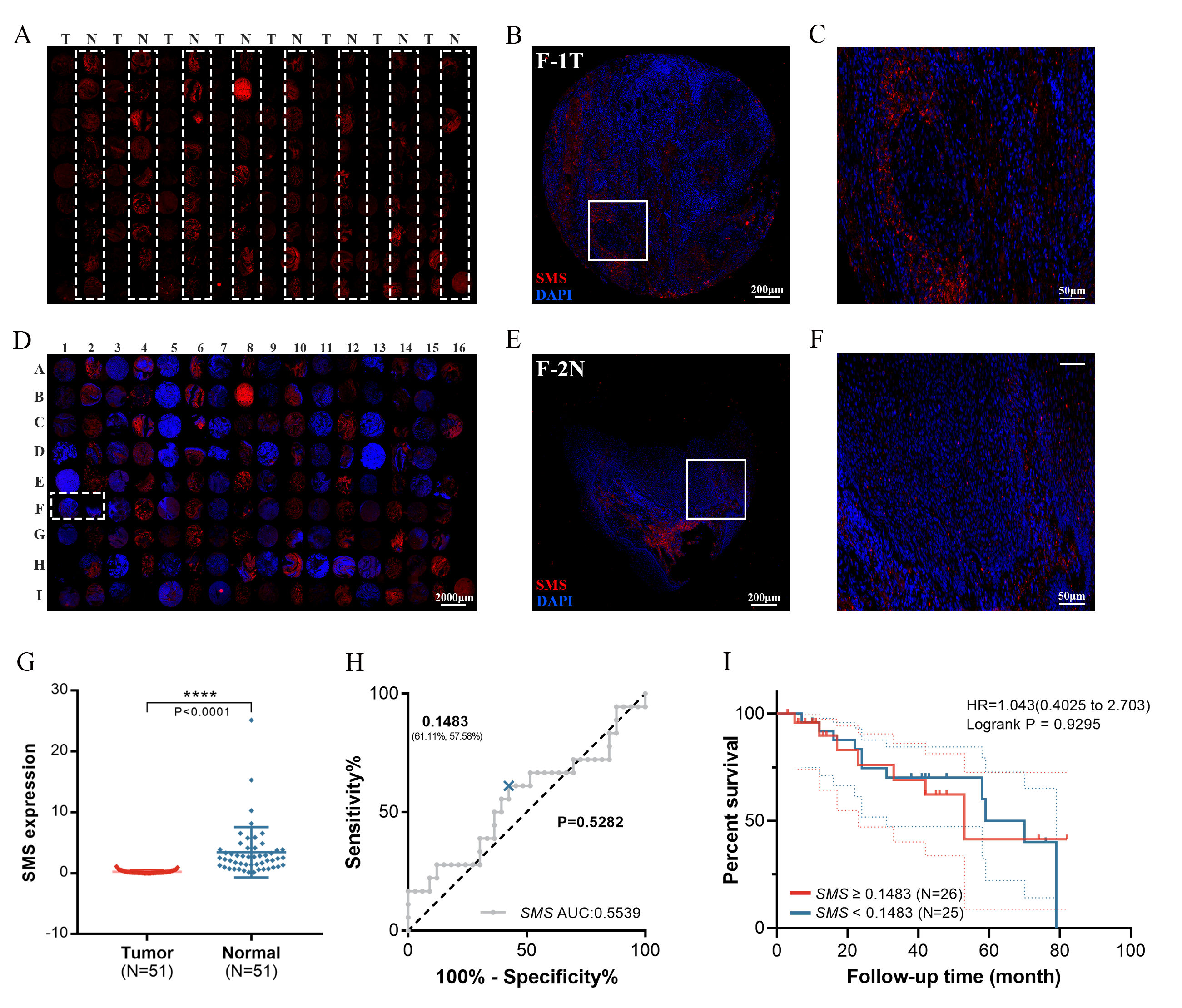

Supplement: Supplementary file 4 — Additional file 4: Figure S4. External validation of SMS expression and prognosis in FDEENT cohort. A SMS immunofluorescence staining on TMA. B, C Merged immunofluorescence staining on F-1 T. D Overview of merged immunofluorescence staining on TMA. E, F Merged immunofluorescence staining on F-2 N. G Quantification of the immunofluorescence signals of SMS. H ROC curve of SMS prediction. I Survival analysis of SMS by the KM plotter in LSCC. [file 12935_2022_2647_MOESM4_ESM.tif]

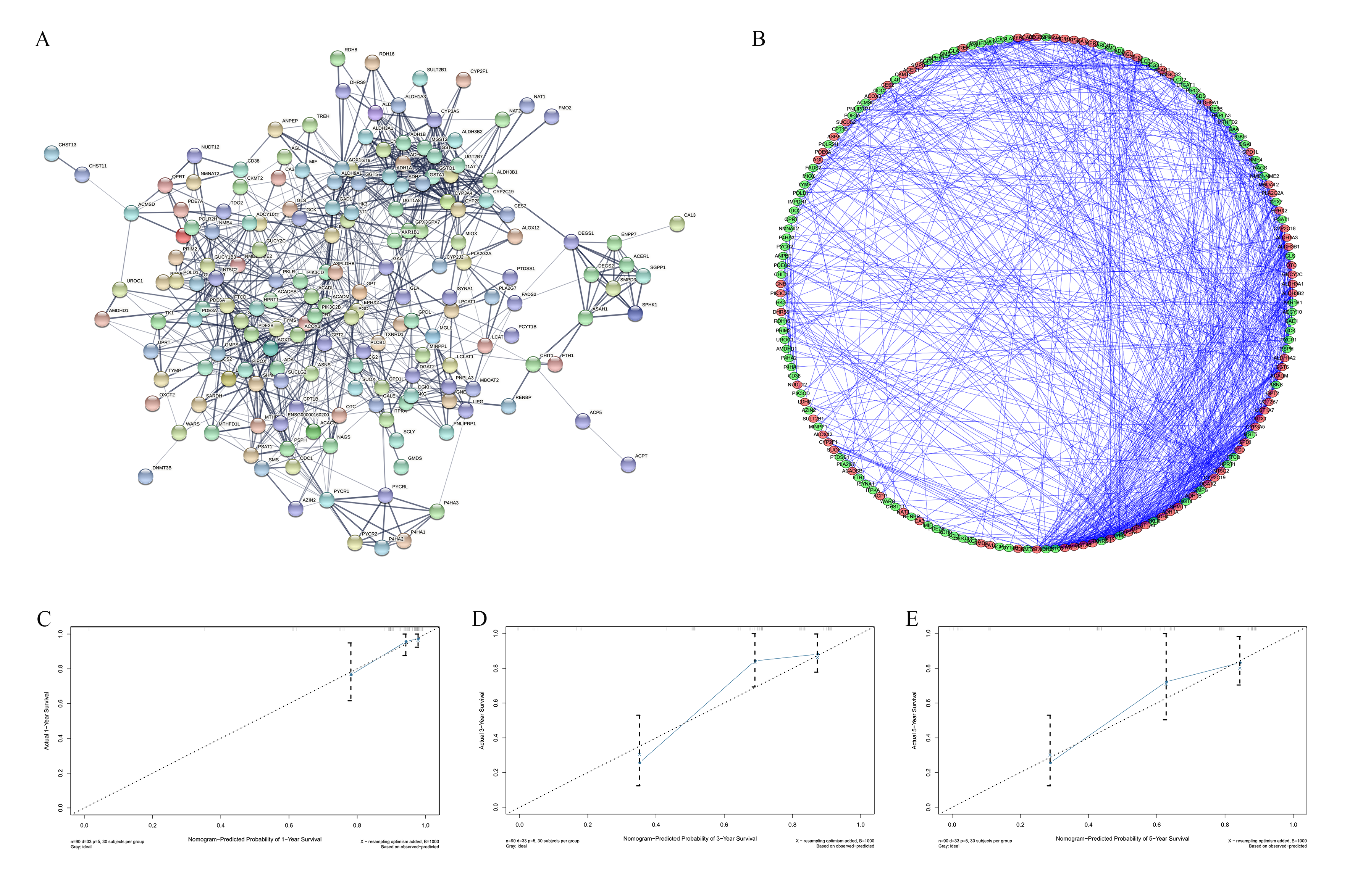

Supplement: Supplementary file 5 — Additional file 5: Figure S5. PPI analysis and verification of the nomogram. A, B Visualization of the PPI network. C, E Calibration curve of prognostic signature for 1-, 3-, and 5-year OS. [file 12935_2022_2647_MOESM5_ESM.tif]

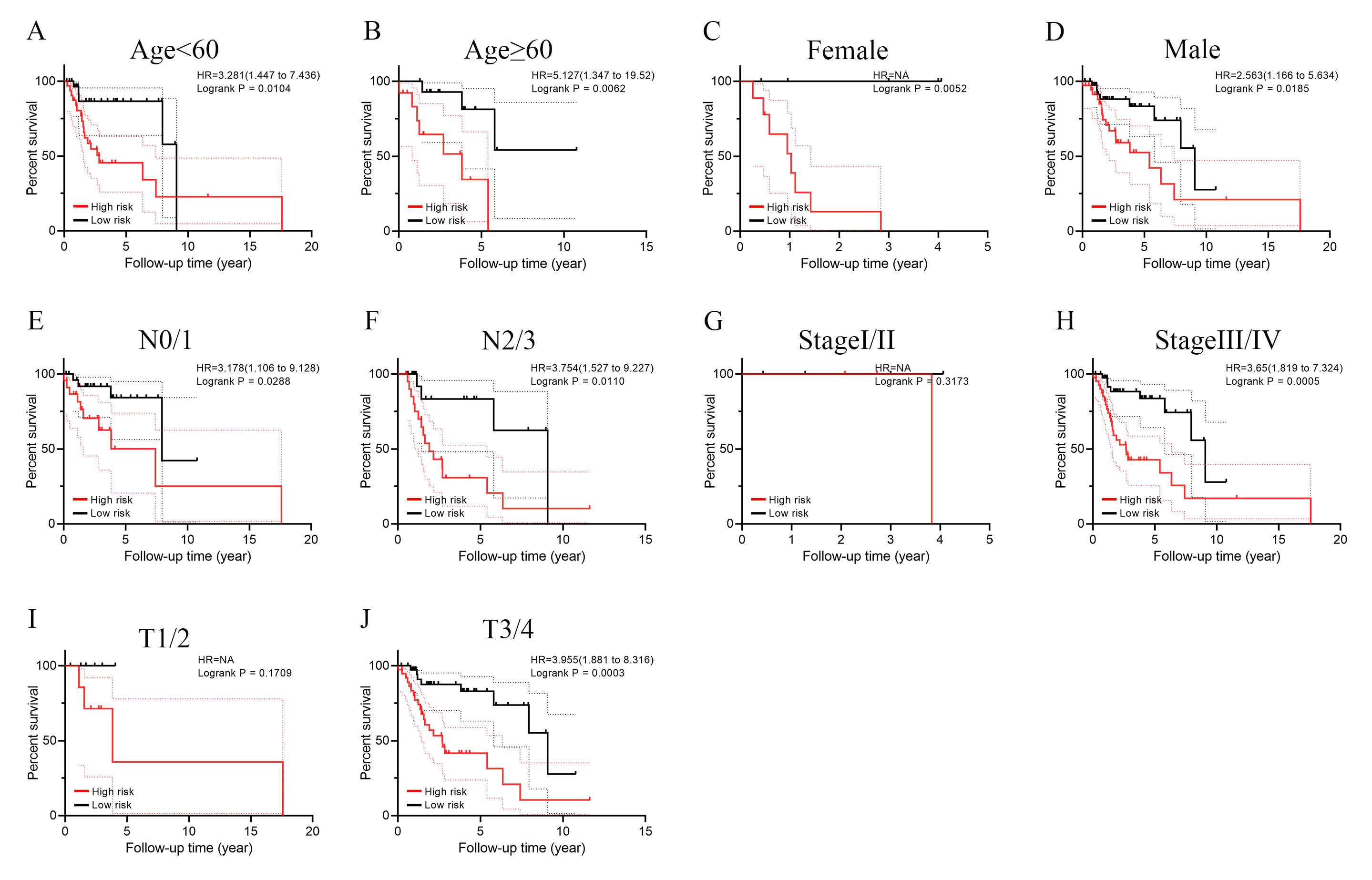

Supplement: Supplementary file 6 — Additional file 6: Figure S6. Kaplan–Meier survival analysis for all LSCC patients according to the prognostic signature stratified by clinicopathological risk factors. A, B The Kaplan–Meier stratified by age. C, D The Kaplan–Meier stratified by gender. E, F The Kaplan–Meier stratified by stage N. G, H The Kaplan–Meier stratified by stage. I, J The Kaplan–Meier stratified by stage T. [file 12935_2022_2647_MOESM6_ESM.tif]
